# Rapid analysis of seed size in *Arabidopsis* for mutant and QTL discovery

**DOI:** 10.1186/1746-4811-7-3

**Published:** 2011-02-08

**Authors:** Rowan P Herridge, Robert C Day, Samantha Baldwin, Richard C Macknight

**Affiliations:** 1 Department of Biochemistry, University of Otago, PO Box 56, Dunedin 9054, New Zealand; 2 New Zealand Institute for Plant and Food Research Ltd, Lincoln, Private Bag 4704, Christchurch 8140, New Zealand

## Abstract

**Background:**

*Arabidopsis thaliana *is a useful model organism for deciphering the genetic determinants of seed size; however the small size of its seeds makes measurements difficult. Bulk seed weights are often used as an indicator of average seed size, but details of individual seed is obscured. Analysis of seed images is possible but issues arise from variations in seed pigmentation and shadowing making analysis laborious. We therefore investigated the use of a consumer level scanner to facilitate seed size measurements in conjunction with open source image-processing software.

**Results:**

By using the transmitted light from the slide scanning function of a flatbed scanner and particle analysis of the resulting images, we have developed a method for the rapid and high throughput analysis of seed size and seed size distribution. The technical variation due to the approach was negligible enabling us to identify aspects of maternal plant growth that contribute to biological variation in seed size. By controlling for these factors, differences in seed size caused by altered parental genome dosage and mutation were easily detected. The method has high reproducibility and sensitivity, such that a mutant with a 10% reduction in seed size was identified in a screen of endosperm-expressed genes. Our study also generated average seed size data for 91 *Arabidopsis* accessions and identified a number of quantitative trait loci from two recombinant inbred line populations, generated from Cape Verde Islands and Burren accessions crossed with Columbia.

**Conclusions:**

This study describes a sensitive, high-throughput approach for measuring seed size and seed size distribution. The method provides a low cost and robust solution that can be easily implemented into the workflow of studies relating to various aspects of seed development.

## Background

More food will need to be produced during the next 50 years than in the entire history of humankind. Therefore, increasing crop yields is a major challenge for the 21^st ^century. Since most of the world's food calories come from seed, one way to meet this challenge is to create plants with more and larger seeds. *Arabidopsis thaliana *is a useful model organism for studying seed development due to its ease of cultivation and extensive genetic and community resources available. Thus far, only a handful of genes are known to be directly involved in determining *Arabidopsis *seed size [1-8].

Genes that regulate seed size can be discovered by screening for mutants or by quantitative trait loci (QTL) analysis to identify the genes that underlie the natural variation in seed size between different accessions. Alonso-Blanco *et al*. [9] performed a QTL analysis on recombinant inbred lines (RILs) from crosses between the small seeded Landsberg *erecta *(L*er*) accession and the large seeded Cape Verde Islands (Cvi) accession. Seed weight and length QTL were mapped as well as those affecting maternal factors that contribute to seed size (such as seed number and leaf size). Six QTL affecting seed size, without significant effects on the maternal plant, were identified [9]. The genes/alleles underlying these QTL have not been determined, which is an important step if they are to be applied in a biotechnological context. Only a few *Arabidopsis *mutants have been identified that directly affect seed size. These mutants reveal that both endosperm and integument growth are involved in seed size determination in *Arabidopsis *[10]. *HAIKU1*, *2 *(*IKU1 *and *2*) and *MINISEED3 *(*MINI3*) act in the same pathway to control early endosperm proliferation and subsequent seed size at maturity [2,4]. *MINI3 *and *IKU2 *are in close proximity to two quantitative trait loci (QTL) discovered by Alonso-Blanco *et al*. [9] suggesting that they may play an important role in the natural variation observed between accessions [4]. SHORT HYPOCOTYL UNDER BLUE1 (SHB1) binds the promoters of *IKU2 *and *MINI3*. However, the *shb1 *mutant only has a minor effect on seed size [7], suggesting that there may be other regulators of seed size upstream of this pathway. The *AUXIN RESPONSE FACTOR2 *(*ARF2*) and *TRANSPARENT TESTA GLABRA2 *(*TTG2*) genes affect seed size via integument cell elongation [5,11]. Crossing *ttg2 *and *iku2 *mutants reveals that, although the genes operate in independent pathways, cross-talk occurs between the integument and endosperm to determine final seed size [10].

Parental genome dosage can also affect seed size. In interploidy crosses, a seed size effect is observed when the ratio of maternal to paternal genomes is altered in the endosperm, with over-representation of paternal genomes resulting in larger seeds whereas the opposite is true for maternal genomes [12]. A similar phenomenon is found when reciprocal crosses of *met1 *mutants are performed, suggesting that DNA methylation plays an important role in seed size determination, via the action of imprinted genes in the endosperm and also hypomethylation in the integuments [13,14].

A major aim of our laboratory is to discover the molecular mechanisms that regulate seed size. We have developed a sensitive, high-throughput method of measuring seed size using a scanner and particle analysis software. Furthermore, by identifying and taking into account certain maternal factors that contribute to seed size we were able to reduce variation, enabling routine detection of subtle differences in size. We show that the approach is capable of identifying differences in seed size due to mutation, parental genome dosage, natural variation and detection of a number of novel seed size QTL.

## Results

### A document scanner and open source image analysis software provide a low cost and reliable means of rapidly measuring seed size

**Figure 1:**
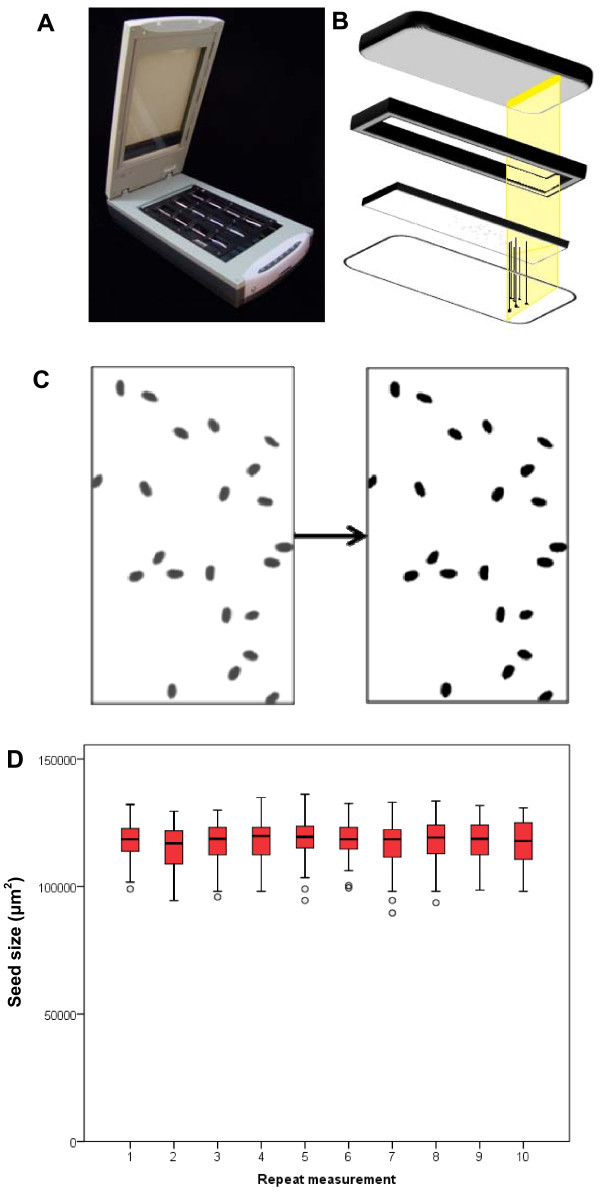
**Reliable measurement of seed size is achieved using transmitted light**. (A) Scanner with slide-holder insert allows simultaneous scanning of 24 samples. (B) Images are generated by emitting light from the lid, through the frame and onto the scanner bed; the seeds cast shadows which are detected by the scanner bed. (C) The resulting images are converted to solid black and white images using the threshold function of ImageJ prior to particle analysis. (D) Ten measurements were made on 54 seeds from a single silique, reorienting the seeds between measurements (circles = outliers > 1.5 IQR from median).

We investigated whether a document scanner could provide a means of rapidly screening a large number of *Arabidopsis *plants for seed size phenotypes with a high degree of sensitivity. Using a commercially available scanner with a resolution of 1200 dpi combined with image analysis software we were able to quickly obtain accurate measurements of seed size. The slide-holder included with the scanner enabled 24 samples to be scanned simultaneously, reducing the amount of labour required (Figure 1A). A major factor with regard to the ease of processing was the use of transmitted light. This enabled us to avoid potential complications due to seed colour or variation in the white background that may be occur when using reflective imaging (Figure 1B). This enabled us to quickly process the images using the "threshold" function of ImageJ and ensured that the resulting black and white images were accurate representations of the seeds (Figure 1C).

Due to the fact that seeds are ellipsoid it is possible that their orientation on the scanner bed will affect the measurement. We tested the ability of the scanner to generate reproducible results from a large number of seeds by measuring the seeds from a single silique multiple times and reorienting the seeds between measurements. The distribution of seed sizes generated by the scanner remained relatively constant (Figure 1D), indicating that the effect of seed orientation is negligible when measuring multiple seeds.

### The biological variation in seed size can be reduced by controlling the growth of the maternal plant

When performing a mutant screen or QTL analysis, variation in seed size must be reduced as much as possible to increase the sensitivity of the screen. More consistent results will allow detection of more subtle phenotypes which may otherwise be overlooked, thus controlling this variation is of great importance. The maternal plant plays an important role in seed size determination; therefore to reduce biological variation in seed size the growth of the maternal plant must be kept constant. We investigated a number of easily controlled factors of the maternal plant that may contribute to variation in seed size.

**Figure 2:**
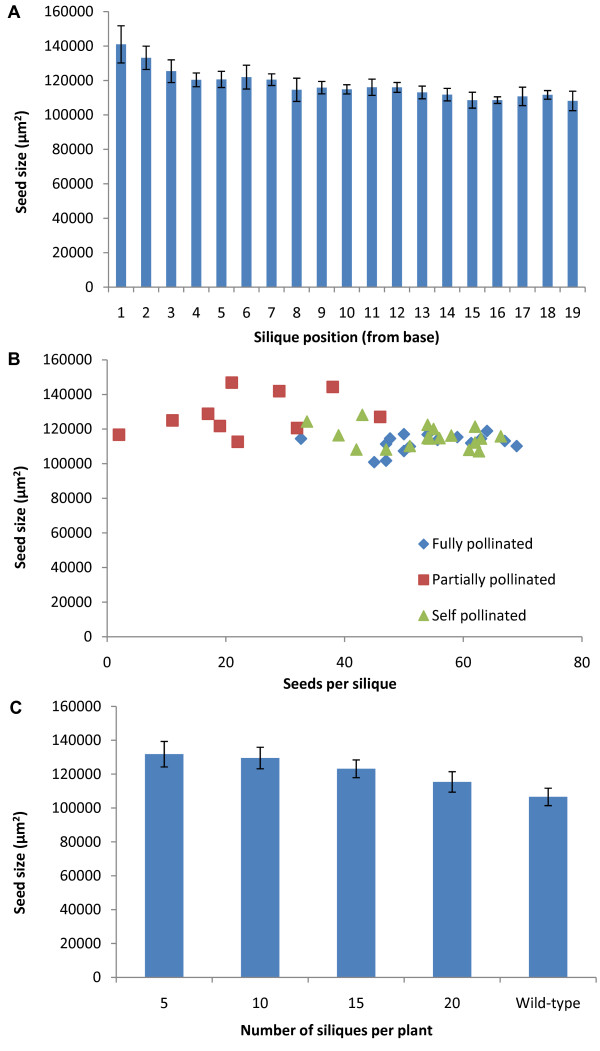
**Factors affecting seed size**. (A) Average seed sizes from siliques in different positions on the main bolt (Error bars = S.D. of biological replicates). (B) Average seed sizes from siliques containing different numbers of seeds after partial, full or self-pollination. (C) Average seed sizes of plants with different numbers of siliques (Wild-type, untrimmed; error bars = S.D. of biological replicates).

To test whether the position of the silique on the main bolt had an effect on seed size, every silique was taken from the main bolt and the seeds measured three times. The first three siliques show a marked increase in seed size, after the fourth silique there is a small decrease in average seed size up the shoot (Figure 2A). The likely cause of the increase in seed size in the first three siliques was the fact that these siliques contained fewer seeds (16, 32 and 45, for silique 1, 2 and 3, respectively, compared with an average of 56 for siliques 4-20). To investigate if the number of seeds in a silique affects seed size, seed number was varied by allowing plants to self-pollinate or emasculating flowers and partially pollinating or fully pollinating the stigma. As the number of seeds in a silique decreases there is some evidence that the average seed size increases, however this is accompanied by an increase in the variation of seed size (Figure 2B). Once a silique contains ~50 seeds this variability is reduced. Thus, fully extended siliques should be selected for seed size measurement to reduce variation. To investigate if the availability of maternal resources affects seed size, the total number of siliques on the plant was altered by trimming auxiliary buds and flowers to allow only a set number of siliques to form. It was found that the more siliques on a plant the smaller the average seed size (Figure 2C). However the standard deviation within treatments was similar between treatments, indicating that there is not an optimum number of siliques for improving accuracy (Figure 2C). The flowering time of a plant may have an effect on seed size due to increased vegetative growth before seed production. To determine the extent to which flowering time affected seed size, plants were grown in short day conditions to delay flowering. Plants were moved into long day conditions once they began to flower to ensure a comparable amount of light was available while producing seeds. The increase in vegetative growth caused by the delayed flowering led to a minor but significant increase in seed size (~10%, p < 0.01, student's T-test; data not shown). Vernalization often reduces the variation in flowering time between different accessions and therefore may be a useful way of increase the likelihood of identifying QTL specifically affecting seed size rather than QTL that influence seed size by affecting maternal resource availability.

Overall, our data highlights the influence maternal factors have on seed size. We concluded that the simplest way to obtain accurate seed size measurements is to remove auxiliary buds to reduce variability in total silique number and take only fully extended siliques from between the fourth and tenth position on the main bolt.

### Validation of the seed size measurements using interploidy crosses and known mutants

**Figure 3:**
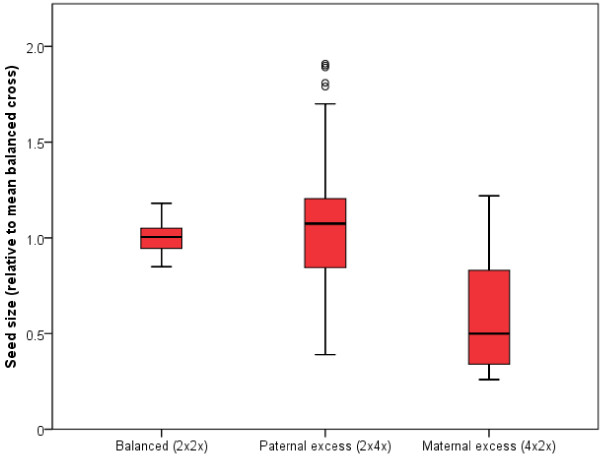
**Parental dosage affects seed size**. Reciprocal crosses were made between diploid and tetraploid Col-0 plants causing a genomic imbalance in the endosperm causing alterations in seed size depending on the direction of the cross. Seeds were measured from the crosses and each measurement was divided by the mean of the balanced cross (2×2×) and plotted on a box and whisker diagram (circles = outliers > 1.5 IQR from median).

Interploidy crosses using C24 and L*er *accessions of *Arabidopsis *produce seeds of variable size, depending on whether there is an excess of maternal or paternal genomes [12]. The average weight of seeds from a 2 × 4× and 4 × 2× cross was ~2.5× and ~0.75× that of a 2×2× cross respectively for both C24 and L*er *accessions [12]. To confirm these results using the Col-0 accession, and to demonstrate the utility of our seed size assay, we performed crosses with diploid and tetraploid Col-0 plants. Surprisingly, we found average seed size to be similar between seeds from a 2×4× and 2×2× cross (See additional file 1: Figure S1.pdf). Similar results were found in interploidy crosses of the L*er *accession (See additional file 1: Figure S1.pdf). It was apparent that a large number of seeds were aborting in the 2×4× cross, thus reducing the average seed size. To better illustrate the differences in seed size between these crosses, measurements from the interploidy crosses were normalized relative to the mean of a balanced cross and plotted on a box and whisker diagram (Figure 3). This showed that although a large proportion of seeds from the 2×4× cross were aborting, there were some that were ~2 fold larger than 2×2× seeds. In addition, although the average size of seeds from a 4×2× cross was significantly less than those of a 2×2× cross, some seeds were still similar in size to those from a balanced cross (Figure 3).

**Figure 4:**
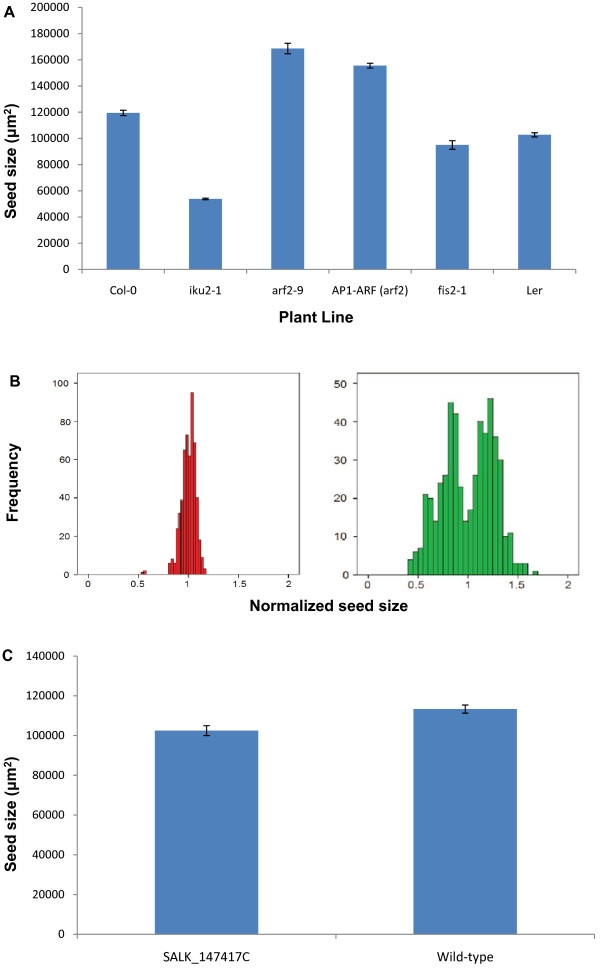
**Known seed size mutants can be detected**. (A) Seeds were measured from *iku2*, *arf2*, *AP1-ARF *(*arf2 *mutant background) and *fis2*. Average seed size was determined and compared to Col-0 and L*er *(Error bars = S.E.M. of biological replicates). (B) Three measurements of seeds from a wild-type (left) and a *fis2-1 *mutant silique (right) were normalized and plotted on a histogram, respectively. (C) Average seed size of SALK_147417C compared to wild-type Col-0 (Error bars = S.E.M. of biological replicates).

To validate the method as a tool for detecting novel seed size mutants we examined a number of known seed size mutants. We grew several seed size mutants under controlled conditions (without auxiliary buds) and measured the seeds in triplicate. Figure 4A shows the average seed size of the *iku2-1*, *arf2-9*, *APETALA1 *promoter driving expression of the *ARF *coding sequence (*AP1-ARF *in an *arf2 *mutant background) and *fis2-1 *mutants. The *iku2-1 *mutant is known to have smaller seeds [2], whereas the *arf2-9 *and *AP1-ARF *(*arf2 *mutant background) mutants have larger seeds [5,15]. The *AP1-ARF *construct rescues the *arf2-9 *mutation in the flowers resulting in improved fertility and a reduced seed size compared to *arf2-9 *mutants [15]. The *fis2-1 *mutant has a 50% rate of seed abortion, where the aborted seeds appear smaller than the viable ones [16]. All mutants were detected with a high level of significance including the subtle difference between *arf2-9 *and *AP1-ARF *mutants (Figure 4A), indicating that the scanner is capable of detecting these known mutants. It is also possible that a mutation may result in an altered distribution of seed sizes within a silique, which can be observed using a histogram. The *fis2-1 *mutant is a model for such a phenotype; seeds of a wild-type silique and a *fis2-1 *mutant silique were measured three times, measurements were normalized by dividing by the average seed area and plotted on histograms (Figure 4B). The 50% seed abortion phenotype is clearly shown in the histogram and suggests that the scanner is capable of identifying mutants based on differences in the distribution of seed sizes in a silique.

To identify novel genes with effects on seed size and demonstrate the effectiveness of the scanner in a high-throughput application we performed a mutant screen. 137 homozygous T-DNA insertion lines from the SALK collection [17] corresponding to 119 endosperm-expressed genes identified by Day *et al*. [18] were selected for the screen. Average seed size for each line was calculated and compared to the average of all other lines grown simultaneously, resulting in a relative increase/decrease in seed size for each line (See additional file 2: Table S1.pdf). Lines that showed a significant (p < 0.05) change in seed size greater than 10% were confirmed by growing alongside wild-type Col-0 plants (data not shown). One line (SALK_147417C) showed a reproducible 10% reduction in seed size (Figure 4C). The identification of a novel mutant as part of a high-throughput screen demonstrates the effectiveness of this method in performing large-scale analysis of seed size phenotypes.

### Variation in seed size between different *Arabidopsis *accessions

**Figure 5:**
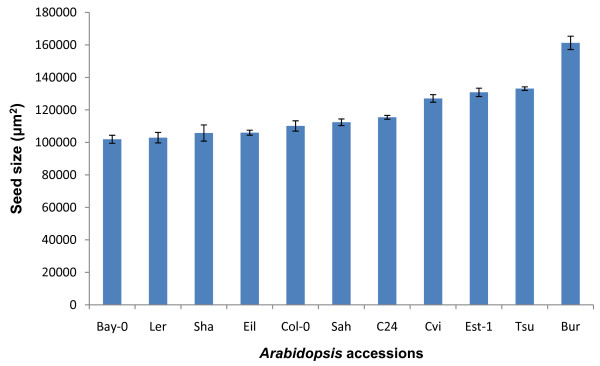
**Variation between accessions can be detected**. Seeds from various accessions were vernalized at 4°C for 3 weeks and final seed size was measured (Error bars = S.E.M. of biological replicates).

A large amount of genetic diversity is present between different accessions of *Arabidopsis*. This genetic diversity can be used in a QTL analysis to discover new loci that regulate seed size. Accessions with the greatest difference in seed size are most informative in a QTL analysis as they are more likely to contain alleles with large and easily detectable effects on seed size. We aimed to detect differences in seed sizes between different accessions of *Arabidopsis *with a view of performing a QTL analysis. Plants were vernalized at 4°C for 3 weeks in an attempt to reduce any differences in seed size caused by flowering time and were grown in controlled conditions (without auxiliary buds). Significant differences in average seed size between accessions were observed (Figure 5). To further validate our method, we also determined the average seed weight of these accessions by weighing a specific number of seeds (~200 for each accession, counted using the scanner and particle analysis software). The average seed weight showed a strong correlation with average seed area (See additional file 3: Figure S2.pdf). Seeds of the Bur accession were clearly the largest, while Bay-0, L*er*, Eil and Sha accessions had the smallest seeds. Although we attempted to reduce biological variation by vernalization and trimming of the auxiliary buds, it is likely that maternal resource allocation still had some influence on the differences we observed. We also examined the seed size of 80 different *Arabidopsis *accessions from the 1001 genomes project [19]. Seeds obtained from the stock center were measured directly on the scanner; the size of seeds from these accessions varied from ~77,000-155,000 μm^2^, a difference of 100% (See additional file 4: Figure S3.pdf). These results provide a basis for analyzing the natural variation in seed size found between accessions.

### Identification of QTL affecting seed size

Based on the differences in seed sizes between accessions (Figure 5), two core populations of 164 RILs resulting from crosses between BurxCol and CvixCol were obtained from INRA [20]. These RILs were selected as Bur had a much larger seed size than Col, and seed size QTL in L*er*xCvi RILs had been mapped previously [9], allowing some comparison with our results. Seeds obtained from the stock center were measured directly using the scanner.

**Figure 6:**
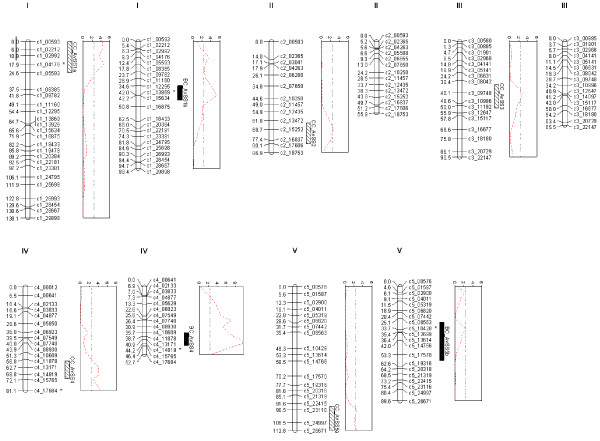
**QTL associated with average seed size (AvSS) for BurxCol (BC) and CvixCol (CC) RIL populations**. Linkage maps are shown for each chromosome with CC on the left and BC on the right. The markers significantly associated with average seed size from single marker analysis are indicated (*). The LOD profiles from interval mapping are shown for each significant QTL including the permuted 5% significance thresholds (dashed lines). QTL identified using multiple QTL modelling are shown by bars and end where the LOD drops by 1.5 for both CC (diagonal) and BC (filled).

Regions of interest were first explored using basic single marker analysis, which revealed significant associations (log of odds (LOD) > 3) with markers on chromosomes 1 and 4 in CvixCol and 1, 4 and 5 for the BurxCol RIL populations (Figure 6). To better define and identify putative QTLs, interval mapping was carried out using the EM algorithm. A 5% significance threshold was calculated as LOD 2.44 and 2.35 for CvixCol and BurxCol, respectively, using 1000 permutations. Based on this threshold, interval mapping identified significant QTL on chromosome 1, 2, 3, 4 and 5 for CvixCol and 1, 4 and 5 for BurxCol (Figure 6). Similar results were obtained using Haley-Knott [21] and the extended Haley-Knott analysis [22] (data not shown). Given that multiple QTL were identified further analysis was used that could better identify and model the effects of multiple QTL segregating in the population. Therefore a 2 QTL analysis (*scantwo*) was used to compare each chromosome for likely additive effect and or interacting (the full model) QTL. The significance of the association was determined using 1000 permutations.

For CvixCol the highest LOD scores were for 2 QTL on chromosomes 1 and 4 with a slightly higher LOD if interactions were allowed (LOD score for the full model of 10.32 versus the additive of 10.28) and the LOD scores were higher for the 2 QTL models versus the single QTL model (5.67). This was followed by an interaction of QTL on chromosomes 2 and 4 (9.28 for 2 QTL model compared to 4.87 for single). There was also weaker evidence for QTL on 3 and 5 based on the full model LOD scores (6.46 compared to 3.6 for just 1 QTL).

For BurxCol the highest LOD scores were also for 2 QTL on chromosomes 1 and 4 (20.11) the full model that included an interaction between the loci was only slightly better than the purely additive model (18.61). There was some evidence of a QTL on 5 with a significant result for the full model (5.61) when compared to chromosome 3 (a chromosome with no large QTL effects). Given this evidence and the results from the linkage mapping a chromosome 5 QTL was also included in the subsequent multiple QTL modeling.

### Multiple QTL modeling

**Figure 7:**
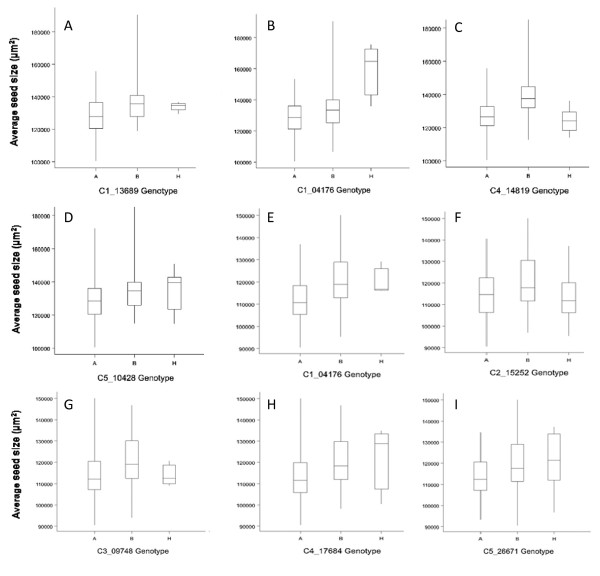
**Boxplots of the average seed size for each QTL marker genotype**. (A-D) A is homozygous for the Col allele, B is homozygous for the Bur allele and H is heterozygous. (E-I) A is homozygous for the Col allele, B is homozygous for the Cvi allele and H is heterozygous.

To further interrogate and to estimate QTL effects, multiple QTL modeling was undertaken. Each putative QTL that had been identified from the interval or 2 QTL analysis was used to develop a multiple QTL model. This was used to determine the amount of variation in seed size explained for both the individual and combined QTL. Various models were tested including all of the QTL identified for the CvixCol and BurxCol crosses, including the two possible QTL on chromosome 4 (as shown by the multiple peaks in Figure 6 from the interval mapping results), individually modeling each of the different positions for the QTL on chromosome 4, and possible interactions identified from the 2 QTL analysis. For CvixCol the model was maximized (LOD 20 and 43% variance explained) when 1 QTL on all chromosomes was tested and the position of the QTL on chromosome 4 was 62.7 cM. The variance explained by the model was 43% with 5 QTL ranging from individual variance of 4% (CC_AvSS3) - 14% (CC_AvSS4). For the BurxCol data the model was slightly improved when a QTL on 5 was included in the model along with the QTL on 1 and 4 (LOD 21 and 44% variance explained). The variance explained by each of the QTL varied from 3.4 (BCAvSS_5) to 15.3% (BCAvSS_4). The variation in average seed size for each genotype at the closest markers to the QTL for both BurxCol and CvixCol RIL populations are shown in Figure 7.

To summarize, in both populations there were major QTLs on chromosomes 1 and 4 with only the QTL on 4 overlapping. Looking at the LOD profiles from the interval mapping using the BurxCol data it is possible that there are actually two regions on chromosome 1 that are important but that the effect of the locus that would overlap CC_AvSS1a is being masked by the effects of the QTL BC_AvSS1b.

## Discussion

Seed size is a trait of considerable importance. However, the small size of seeds and high levels of biological variation hinder its study in *Arabidopsis*. Here, we describe a rapid method of measuring seed size that is capable of detect subtle differences. This method offers advantages over weighing large numbers of seeds to determine seed size, as it avoids the need to count individual seeds and, as every seed is measured individually, alterations in the distribution of seed sizes is easily identified. We demonstrate the utility of this method by successfully using it to identify a seed size mutant and seed size QTL.

One important determinant of seed size is the rate and duration of endosperm proliferation during the early stages of seed development. This has been demonstrated in crosses between *Arabidopsis *plants of different ploidies [12]. Crosses between a diploid seed parent and a tetraploid pollen parent produces seeds that are more than double the weight of seeds from 2×2× crosses and over 40% heavier than those from 4×4× crosses; these large seeds also contain large embryos [12]. Seed development in these crosses is characterized by an increase in the rate and duration of division in peripheral endosperm, delayed endosperm cellularization and an increase in the size of chalazal endosperm [12]. Our analysis of interploidy crosses using the Col-0 accession gave a slightly different result whereby seeds from 2×4× crosses had a tendency to abort - as was seen in 2×6× crosses using the C24 accession [12]. However, in addition to the aborting seeds several seeds were ~2-fold larger than the average 2×2× seed, a result which is similar to those reported by Scott *et al*. [12]. Additionally, we identified the presence of some seeds resulting from a 4×2× cross which were similar in size to 2×2× seeds (Figure 3). A more recent study [23] has shown that seed abortion in interploidy crosses is dependent on the accession of *Arabidopsis *used, and that when Col-0 is used as the male parent in a 2×4× cross seed abortion occurs at a high rate, consistent with the results we obtained. Parental genome dosage effects, including the dosage of imprinted genes, have been implicated in seed viability of interploidy crosses [24]; however, it is known that the *TTG2 *gene, expressed in the maternal sporophytic tissues, plays a role in seed viability of these crosses and that the Col allele of *TTG2 *has a negative impact on seed viability in interploidy crosses [23].

The role of the endosperm in determining seed size was also revealed by three mutants that function in the same genetic pathway, *HAIKU1 (IKU1), IKU2 *and *MINISEED3 (MINI3) *[2,4]. The small seed size of these mutants is the result of reduced growth and early cellularization of the endosperm. As a first step to identify additional genes involved in endosperm development, we obtained the endosperm transcriptome from laser dissected proliferating endosperm tissue [18]. We identified 800 genes that were preferentially expressed during early endosperm development. To investigate if any of these genes affect endosperm proliferation, and therefore final seed size, we screened through homozygous SALK T-DNA insertion lines in these genes [17]. Using the scanner and image software allowed us to rapidly measure the seed size of 137 T-DNA lines. This identified a mutant with ~10% increase in seed size, which had an insertion in exon 3 of the At2g01810 locus (Figure 4C; Additional file 2: Table S1.pdf). Two other T-DNA insertion lines were also analyzed for this gene, SALK_099086C in exon 3 and a segregating line, SALK_079456, in exon 1. However, these lines did not show any effect on seed size (See additional file 2: Table S1.pdf), suggesting that the seed size effect seen in SALK_147417C was possibly due to another mutation. Further analysis of this mutant indicated a chromosomal translocation had occurred which may be causing the phenotype (data not shown), so a map-based cloning approach will be needed to isolate the mutation. Nonetheless, the discovery of this seed size mutant demonstrates that our method is effective way of screening for novel mutants.

Another approach for identifying genes involved in determining seed size is to utilize the considerable natural variation in the size of seeds from different *Arabidopsis *accessions. We grew 11 accessions under controlled conditions and measured the seed size, identifying the Bur accession as having the largest seeds (Figure 5). An additional 80 accessions with genome sequences available from the 1001 genomes project [19] were measured directly using seeds obtained from the stock center (See additional file 4: Figure S3.pdf). The results obtained in our study are similar to those reported by de Jong *et al*. [25] who measured average seed weight of 24 accessions. Accessions with large differences in seed size offer an ideal resource for identifying the underlying genetics. To determine if our method for measuring seed size could be used to identify QTL, we obtained two RIL populations generated using Col and the large seed size accessions Cvi and Bur. As we were interested in discovering QTL with a major affect on seed size, we limited our analysis to the core populations of 164 RIL lines, described in Simon *et al*. [20]. Five QTL were identified from the CvixCol RIL population and four QTL from the BurxCol RIL population. These QTL explained ~44% of the variation in seed size. The QTL with the largest effect (explaining ~15% of the seed size variation) mapped to a similar location on chromosome 4 in both RIL populations. A major QTL in this position was also identified by Alonso-Blanco *et al*. [9] in CvixLer RILs. Interestingly, this QTL region does not encompass the chromosome 4 genes, *AINTEGUMENTA *or *APETALA2*, known to affect seed size [1,3]. The CvixL*er *QTL analysis of Alonso-Blanco *et al*. [9] showed that the entire lower arm of chromosome 4 had an effect on seed size, suggesting a number of genes with the potential to affect seed size might be located within this region. The QTL region of chromosome 5 segregating in the CvixCol RIL population contains the gene for *ARF2*, raising the possibility that this gene may be causing differences in seed size between the Cvi and Col accessions. A QTL in a similar position on chromosome 5 was also detected by Alonso-Blanco *et al*. [9] suggesting that the Cvi allele in this position is likely to have a strong effect on seed size. However, we did not detect the major QTL on chromosome 1 identified by Alonso-Blanco *et al*. [9], and thought to correspond to *MINI3 *[4], suggesting that the Col, Bur and Cvi alleles of this QTL have a similar effect on seed size. We are currently backcrossing a number of the RILs with Col to generate near isogenic lines to facilitate the cloning of the major QTL using a map-based approach. The fact that the only the chromosome 4 QTL mapped to similar region in the two RIL populations, highlights the importance of using multiple parents to identify different alleles capable of affecting seed size. With an increasing number of RIL populations becoming available [20,26,27], our method for rapidly and accurately determining the seed size of the individual RILs should help facilitate the discovery of novel QTL.

## Conclusions

We have developed a simple, inexpensive and rapid method for measuring seed size that offers significant advantages over measuring seed weight. Using this method, we identified a mutant with smaller seeds and discovered a number of seed size QTL, thus proving its utility in high-throughput and large scale applications.

## Methods

### Plant material and growth conditions

All wild-type diploid Arabidopsis accessions were obtained from the Arabidopsis Biological Resource Centre (Ohio State University, USA). The *iku2-1 *[2] and *fis2-1 *[16] mutants were provided by Dr Ming Luo (CSIRO, Black Mountain, AUS) and the *arf2 *[5], *AP1-ARF *(in *arf2 *background) mutants [15] and tetraploid Col-0 were supplied by Professor Rod Scott (University of Bath, UK). Plants were sown on 1/2 MS media grown at 20°C with a 16 h photoperiod (8 h for short day plants) with light levels of ~100 μE.m^2^.s^-1^. Seedlings were transferred to potting mix after ~2 weeks on 1/2 MS media.

### Seed size measurement

Siliques were harvested once they had turned completely brown but before they had dropped seeds. Siliques were allowed to dry in open microcentrifuge tubes for at least three days before measurement. Dried silique material was removed using forceps and the seeds were spread onto the scanner bed (Microtek Scanmaker i800) ensuring that no seeds were touching. Images were taken of each individual silique at a resolution of 1200 dpi using transmitted light. ImageJ particle analysis software was used to measure seed area [28]. Images were processed using the "threshold" feature of ImageJ (to an arbitrary value of 162 on the greyscale) and seed size was measured using the "particle analysis" feature, with a lower limit 30,000 μm^2 ^to exclude any non-seed material. Data was analyzed using Microsoft Excel and SPSS statistical analysis software.

### Manual pollination

Flowers were emasculated using fine-tipped forceps taking care not to damage the ovary. Two days after emasculation, pollen from the appropriate male parent was applied to the tip of the stigma.

### QTL analysis

Linkage maps were reconstructed for both populations using marker and recombination data from VNAT [29]. QTL analysis was carried out with the R/qtl package [30,31] and executed in R [32]. The linkage maps were initially assessed for associations with average seed size using single marker analysis and by then interval mapping using the EM algorithm. LOD thresholds were calculated using 1000 permutations for a significance level of 5%. A two-QTL genome scan using *scantwo *analysis was then used to identify QTL with additive or interactive effects (referred to as the full model) and significance thresholds were determined using 1000 permutations. The results from these analyses were used to develop multiple QTL models that were compared using the *makeqtl *and *fitqtl *functions. The best model was chosen based on LOD score, %variance explained and simplicity. This model was then used to determine improved estimates of the QTL locations using *refineqtl*. The 1.5 LOD intervals were then calculated for each individual QTL. Each QTL was given a name based on the population CC (CvixCol) or BC (BurxCol), AvSS (for average seed size), chromosome (number) and individual QTL identifier (letter) where the position along the chromosome was different. The linkage maps and QTL summary were constructed using MapChart v2.2 [33].

## Competing interests

The authors declare that they have no competing interests.

## Authors' contributions

RCD instigated the use of transmitted light for particle analysis of seed size and both RCD and RCM advised RPH on study design. RPH developed the approach and carried out the validation experiments. SB performed the QTL analysis. RPH wrote the manuscript with contributions from RCD, SB and RCM. All authors read and approved the final manuscript.

## Supplementary Material

Additional file 1 **Average seed sizes of interploidy crosses of Columbia and Landsberg *erecta *accessions**. Click here for file

Additional file 2 **Relative seed size of T-DNA insertion lines**. Click here for file

Additional file 3 **Correlation between average seed area and average seed weight for various accessions of *Arabidopsis***. Click here for file

Additional file 4 **Average seed sizes of 80 accessions from the 1001 genomes project**. Click here for file
