# Supplementary material for: Rapid analysis of seed size in Arabidopsis for mutant and QTL discovery
Source: Plant Methods. 2011 Feb 8;7:3. doi: 10.1186/1746-4811-7-3 (PMC3046896; doi:10.1186/1746-4811-7-3)
Supplement: Additional file 1 — Average seed sizes of interploidy crosses of Columbia and Landsberg erecta accessions. [file 1746-4811-7-3-S1.PDF]

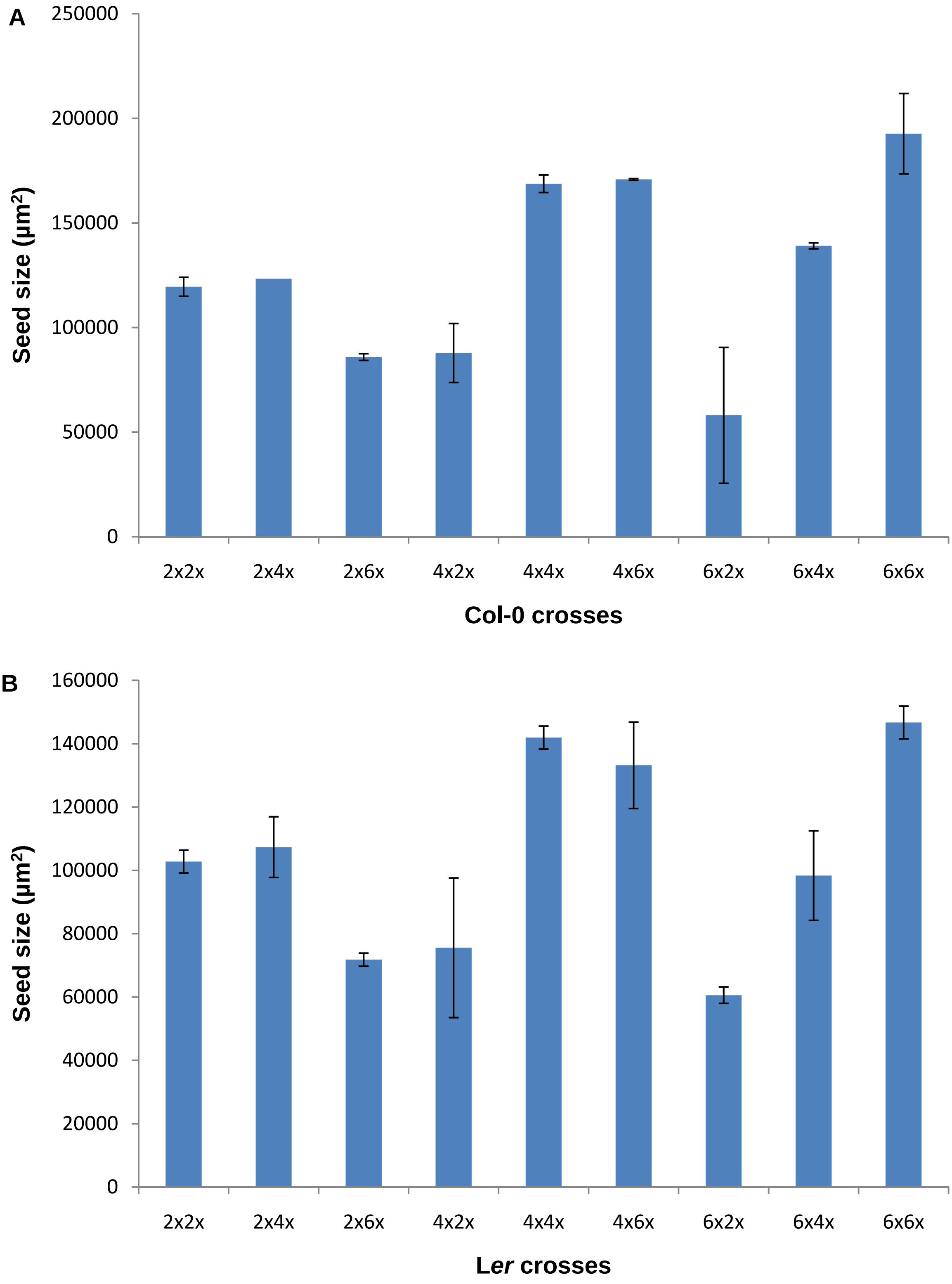

Figure S1. Average seed sizes of interploidy crosses

Crosses between diploid, tetraploid and hexaploid *Arabidopsis* plants were performed for Columbia (A) and Landsberg *erecta* (B) and seed size was measured in triplicate (Error bars = S.D. of biological replicates)
