# Supplementary material for: Rapid analysis of seed size in Arabidopsis for mutant and QTL discovery
Source: Plant Methods. 2011 Feb 8;7:3. doi: 10.1186/1746-4811-7-3 (PMC3046896; doi:10.1186/1746-4811-7-3)
Supplement: Additional file 2 — Relative seed size of T-DNA insertion lines. [file 1746-4811-7-3-S2.PDF]

Table S1. Relative seed size of T-DNA insertion lines

Locus[a]	SALK line	Seed size (relative to mean)[b]	P-value[c]
At2g01810[d]	SALK_147417C	0.85	0.00
At5g59810	SALK_025087C	0.87	0.00
At5g56200	SALK_149548C	0.88	1.00
At5g58810	SALK_119237C	0.88	1.00
At4g11400	SALK_016155C	0.89	0.00
At1g44130	SALK_045883C	0.90	1.00
At5g24316	SALK_093776C	0.90	0.02
At2g46140	SALK_149928C	0.91	0.08
At5g42370	SALK_047812C	0.91	0.00
At2g24740	SALK_123140C	0.92	0.47
At5g17320	SALK_079210C	0.92	0.00
At1g02980	SALK_012144C	0.92	0.01
At2g32340	SALK_059433C	0.92	0.01
At5g10170	SALK_120131C	0.92	0.54
At5g10440	SALK_127016C	0.92	0.00
At5g15140	SALK_102165C	0.92	1.00
At1g44130	SALK_152311C	0.93	0.76
At2g28880	SALK_095283C	0.93	0.00
At5g07210	SALK_005772C	0.94	0.26
At1g03130	SALK_011908C	0.94	0.03
At1g06020	SALK_122966C	0.94	0.09
At2g27070	SALK_042719C	0.94	1.00
At2g42090	SALK_045116C	0.94	0.00
At5g46810	SALK_044084C	0.94	0.05
At5g59190	SALK_149055C	0.94	0.74
At5g10440	SALK_085720C	0.95	1.00
At5g15050	SALK_117005C	0.95	1.00
At5g40260	SALK_092654C	0.95	0.78
At5g54270	SALK_005168C	0.95	1.00
At1g08980	SALK_019823C	0.96	1.00
At1g13680	SALK_048820C	0.96	1.00
At1g50950	SALK_011611C	0.96	1.00
At1g71230	SALK_007134C	0.96	1.00
At2g06520	SALK_011554C	0.96	0.31
At2g32360	SALK_034316C	0.96	1.00
At3g24790	SALK_152499C	0.96	1.00
At4g14780	SALK_029496C	0.96	1.00
At4g30090	SALK_123089C	0.96	1.00
At5g13200	SALK_017675C	0.96	1.00
At1g49790	SALK_009895C	0.97	1.00
At1g79370	SALK_090140C	0.97	1.00
At2g03190	SALK_047421C	0.97	0.00
At2g35260	SALK_031802C	0.97	1.00

Locus[a]	SALK line	Seed size (relative to mean)[b]	P-value[c]
At5g22980	SALK_136908C	0.97	1.00
At1g05160	SALK_136249C	0.98	1.00
At1g08065	SALK_097331C	0.98	1.00
At1g14080	SALK_099500C	0.98	1.00
At1g15040	SALK_031983C	0.98	1.00
At1g18730	SALK_056498C	0.98	1.00
At1g18730	SALK_095654C	0.98	1.00
At2g37750	SALK_016668C	0.98	1.00
At5g42370	SALK_019700C	0.98	1.00
At5g58840	SALK_052517C	0.98	1.00
At5g66150	SALK_055471C	0.98	1.00
At1g09550	SALK_061326C	0.99	1.00
At1g50950	SALK_009396C	0.99	1.00
At2g20290	SALK_132190C	0.99	1.00
At2g24810	SALK_040322C	0.99	1.00
At2g34990	SALK_025813C	0.99	1.00
At4g02780	SALK_027931C	0.99	1.00
At4g13090	SALK_032765C	0.99	1.00
At4g39650	SALK_069311C	0.99	1.00
At5g14740	SALK_042597C	0.99	1.00
At5g20440	SALK_053800C	0.99	1.00
At5g38450	SALK_093028C	0.99	1.00
At5g65890	SALK_072160C	0.99	1.00
At3g66656	SALK_042875C	1.00	1.00
At1g03130	SALK_125057C	1.00	1.00
At2g24640	SALK_136941C	1.00	1.00
At2g24640	SALK_137176C	1.00	1.00
At2g26050	SALK_113297C	1.00	1.00
At2g26880	SALK_150116C	1.00	1.00
At2g46140	SALK_149092C	1.00	1.00
At3g25990	SALK_095404C	1.00	1.00
At5g05250	SALK_071767C	1.00	1.00
At5g15050	SALK_080923C	1.00	1.00
At5g61260	SALK_046895C	1.00	1.00
At1g10680	SALK_050729C	1.01	1.00
At1g78500	SALK_060682C	1.01	1.00
At4g13080	SALK_061055C	1.01	1.00
At5g05760	SALK_057421C	1.01	1.00
At5g08240	SALK_138507C	1.01	1.00
At5g53840	SALK_046367C	1.01	1.00
At5g59190	SALK_075909C	1.01	1.00
At2g01810[d]	SALK_099086C	1.02	1.00
At1g16980	SALK_009815C	1.02	1.00
At1g19150	SALK_132257C	1.02	1.00

Locus[a]	SALK line	Seed size (relative to mean)[b]	P-value[c]
At1g34410	SALK_020702C	1.02	1.00
At1g65760	SALK_003055C	1.02	1.00
At2g35260	SALK_058830C	1.02	1.00
At3g28850	SALK_070907C	1.02	1.00
At3g55590	SALK_111405C	1.02	1.00
At4g30380	SALK_005318C	1.02	1.00
At1g78710	SALK_012450C	1.03	1.00
At4g18910	SALK_126593C	1.03	1.00
At5g40430	SALK_063356C	1.03	1.00
At5g45680	SALK_023405C	1.03	1.00
At2g20595	SALK_063881C	1.04	1.00
At3g26310	SALK_118797C	1.04	1.00
At4g26260	SALK_027238C	1.04	1.00
At5g22740	SALK_075579C	1.04	1.00
At5g40710	SALK_087836C	1.04	1.00
At5g53520	SALK_033058C	1.04	1.00
At1g30710	SALK_112240C	1.05	1.00
At1g54280	SALK_150173C	1.05	1.00
At1g62780	SALK_047296C	1.05	0.26
At1g74870	SALK_032593C	1.05	1.00
At2g20170	SALK_053076C	1.05	1.00
At3g30540	SALK_122701C	1.05	0.01
At4g30380	SALK_110534C	1.05	0.79
At4g32080	SALK_043689C	1.05	0.07
At5g05220	SALK_097371C	1.05	0.02
At5g25950	SALK_029819C	1.05	1.00
At1g34410	SALK_121828C	1.06	1.00
At1g64290	SALK_011135C	1.06	1.00
At3g01880	SALK_023463C	1.06	0.30
At3g19580	SALK_132562C	1.06	1.00
At3g47470	SALK_138555C	1.06	1.00
At5g45680	SALK_047208C	1.06	1.00
At1g68590	SALK_104063C	1.07	0.50
At1g73480	SALK_095195C	1.07	1.00
At2g03190	SALK_046141C	1.07	1.00
At2g44560	SALK_142147C	1.07	1.00
At3g49520	SALK_009123C	1.07	1.00
At1g22670	SALK_102113C	1.08	0.02
At1g78060	SALK_024639C	1.08	0.32
At2g24640	SALK_135277C	1.08	0.32
At2g39980	SALK_113684C	1.08	0.01
At5g09370	SALK_128996C	1.08	0.01
At5g09730	SALK_078171C	1.08	0.03
At1g75600	SALK_137736C	1.10	0.00

Locus[a]	SALK line	Seed size (relative to mean)[b]	P-value[c]
At2g20290	SALK_018032C	1.10	0.14
At3g18080	SALK_025725C	1.11	0.00
At5g26630	SALK_033801C	1.11	1.00
At1g44575	SALK_095156C	1.12	0.21
At2g42220	SALK_045769C	1.13	0.30

[a] TAIR locus ID

[b] Seed size of individual lines was compared to the mean of all other lines grown simultaneously (mean of line/mean of all lines grown simultaneously)

[c] P-values were calculated by comparing the average seed size of siliques from each line to the average of all siliques using a student's t-test, and adjusted using the Bonferroni correction for multiple comparisons

[d] Another segregating line corresponding to the gene At2g01810 was analysed (SALK_079456). Plants carrying the insertion had an average seed size of 1.03 (p = 0.41, student's t-test) relative to those without.
