# Supplementary material for: Rapid analysis of seed size in Arabidopsis for mutant and QTL discovery
Source: Plant Methods. 2011 Feb 8;7:3. doi: 10.1186/1746-4811-7-3 (PMC3046896; doi:10.1186/1746-4811-7-3)
Supplement: Additional file 3 — Correlation between average seed area and average seed weight for various accessions of Arabidopsis. [file 1746-4811-7-3-S3.PDF]

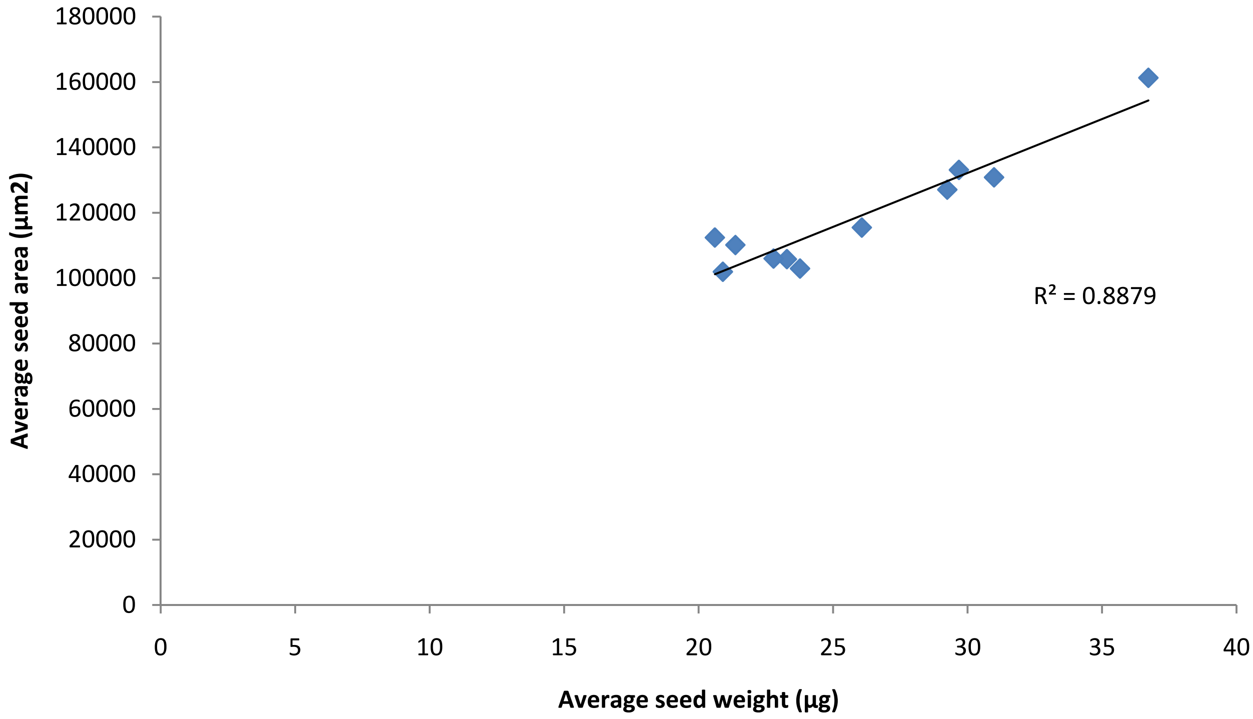

Figure S2. Correlation between average seed area and average seed weight for various accessions of *Arabidopsis*

Seeds from 11 natural accessions of *Arabidopsis* were weighed and average seed weight determined. This data was plotted against average seed area (see fig. 5) and a linear trend-line was fitted.
