# Supplementary material for: Rapid analysis of seed size in Arabidopsis for mutant and QTL discovery
Source: Plant Methods. 2011 Feb 8;7:3. doi: 10.1186/1746-4811-7-3 (PMC3046896; doi:10.1186/1746-4811-7-3)
Supplement: Additional file 4 — Average seed sizes of 80 accessions from the 1001 genomes project. [file 1746-4811-7-3-S4.PDF]

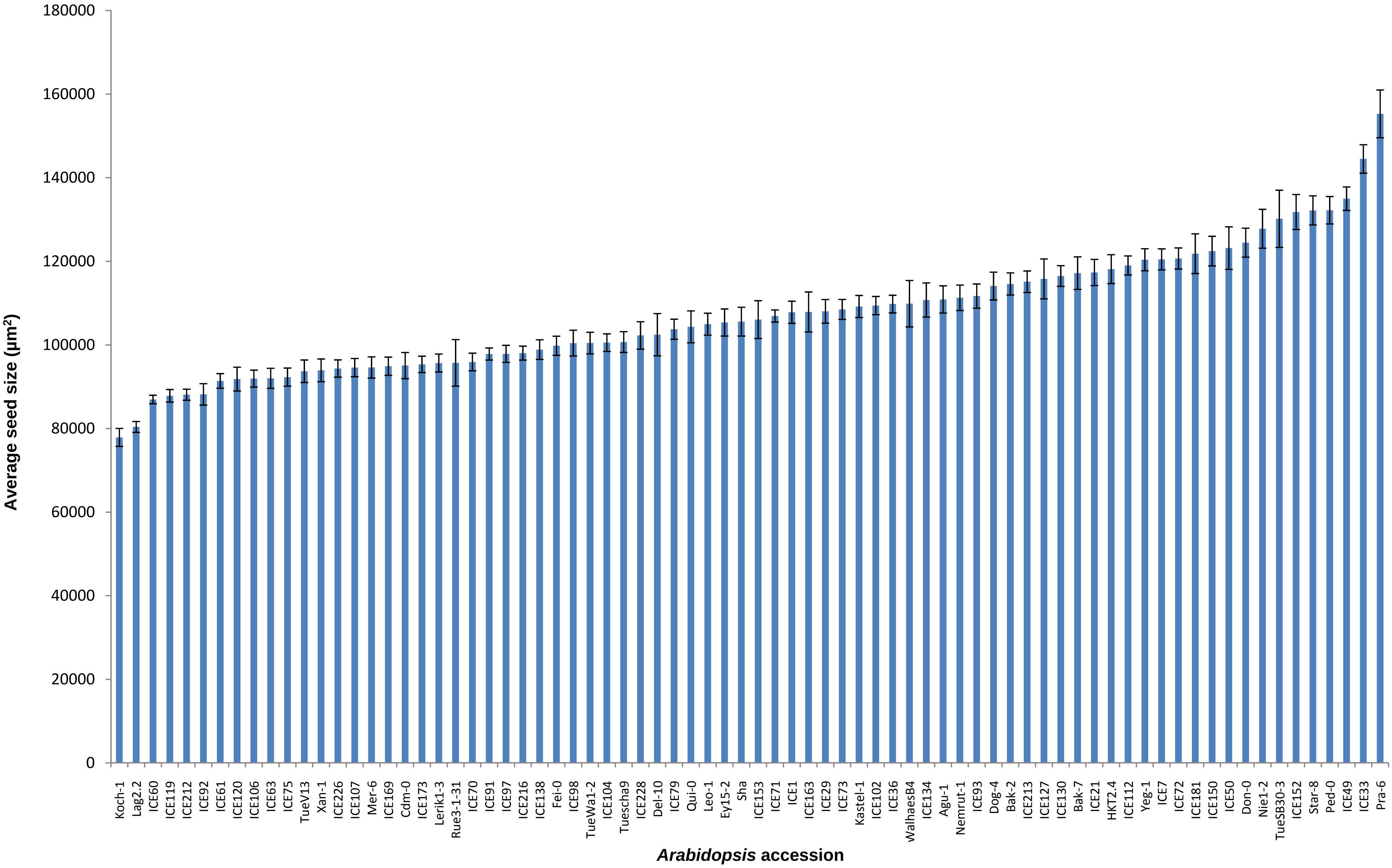

Figure S3. Average seed sizes of 80 natural accessions from the 1001 genomes project

Seeds were measured directly from the stock centre on the scanner. (Error bars = S.E.M.)
